# Analysis of Force Sensing Accuracy by Using SHM Methods on Conventionally Manufactured and Additively Manufactured Small Polymer Parts

**DOI:** 10.3390/polym14183755

**Published:** 2022-09-08

**Authors:** Alireza Modir, Ibrahim Tansel

**Affiliations:** Department of Mechanical and Materials Engineering, Florida International University, Miami, FL 33174, USA

**Keywords:** convolutional neural network, additive manufacturing, ABS, SHM

## Abstract

Fabricating complex parts using additive manufacturing is becoming more popular in diverse engineering sectors. Structural Health Monitoring (SHM) methods can be implemented to reduce inspection costs and ensure structural integrity and safety in these parts. In this study, the Surface Response to Excitation (SuRE) method was used to investigate the wave propagation characteristics and load sensing capability in conventionally and additively manufactured ABS parts. For the first set of the test specimens, one conventionally manufactured and three additively manufactured rectangular bar-shaped specimens were prepared. Moreover, four additional parts were also additively manufactured with 30% and 60% infill ratios and 1 mm and 2 mm top surface thicknesses. The external geometry of all parts was the same. Ultrasonic surface waves were generated using three different signals via a piezoelectric actuator bonded to one end of the part. At the other end of each part, a piezoelectric disk was bonded to monitor the response to excitation. It was found that hollow sections inside the 3D printed part slowed down the wave travel. The Continuous Wavelet Transform (CWT) and Short-Time Fourier Transform (STFT) were implemented for converting the recorded sensory data into time–frequency images. These image datasets were fed into a convolutional neural network for the estimation of the compressive loading when the load was applied at the center of specimens at five different levels (0 N, 50 N, 100 N, 150 N, and 200 N). The results showed that the classification accuracy was improved when the CWT scalograms were used.

## 1. Introduction

In recent years, additive manufacturing (AM) applications have grown rapidly to produce complex parts that cannot be manufactured with traditional methods. Manufacturing using additive manufacturing lowers the manufacturing costs, saves energy and materials, and eliminates the tooling costs [1,2]. One of the most commonly used additive manufacturing techniques is fused filament fabrication, also known as fused deposition modeling (FDM). The internal geometry of AM parts can be customized with repetitive patterns, called infills, which can help to reduce weight, material, and manufacturing time [3]. As AM parts become increasingly popular in many engineering sectors, structural health monitoring (SHM) methods will be required for assessing the structural integrity and detecting damage in these parts [4,5,6]. As part of the SHM approaches, damage identification methods are used to monitor a wide range of structural faults and external loading applied to the structures. Since additively manufactured polymer parts have a small size and a high attenuation, it is necessary to adjust the current SHM techniques accordingly.

Surface (Rayleigh) waves on solid materials can travel long distances, and their characteristics change when they pass sections with cracks, corrosion, loose bolts, or external loads [7]. By using SHM methods that are based on Lamb waves, it is possible to detect the faults originating from a change in wave characteristics or echoes. There are studies in the literature implementing SHM methods for the quality control of additive manufacturing parts [8,9]. The existence of hidden geometries such as multiple skin thicknesses, infill patterns, and infill ratios can vary the specifications of wave propagation [10,11]. In previous studies, sine and sweep sine waves were extensively used as excitation signals. Fast Fourier Transform (FFT) [12], Wavelet [13], Cepstrum [14], and Mode Decomposition [15] methods were used for the analysis of the monitored signals. A new excitation signal consisting of multiple pulses with different widths was introduced in this study, which eliminated the need for a signal generator and could be produced using only digital electronic circuits.

Continuous monitoring of the structural condition using SHM methods requires extensive data collection and analysis. Artificial intelligence (AI) techniques have been widely used for the interpretation of data [16]. Classical machine learning (ML) classifiers such as Support Vector Machines (SVM) [17,18], Artificial Neural Networks (ANN) [19,20], k-means clustering [21,22], and Principal Component Analysis (PCA) [23,24] were used to map the patterns of sensory data for the detection and classification of damages and defects [25]. In a study performed by Zhang et al. [26], SVM was used for classifying different types of damage in an aluminum beam. Khatir et al. [27] investigated damage detection and quantification in a laminated composite plate using ANN. Bouzenad et al. [28] employed the k-mean algorithm for the condition monitoring of pipelines. There are several challenges associated with traditional machine learning techniques, including the handling of large datasets, the determination of features, and the selection of features that are damage-sensitive [29]. In contrast, Deep Learning (DL) methods do not require feature selection for classification even when they are used to classify very complex datasets such as pictures [30] nor do they require human intervention for distinguishing multiple classes with high accuracy [31]. The two-dimensional Convolutional Neural Network (CNN) is a very widely used Deep Learning (DL) algorithm that consists of three layers of neurons (input layer, hidden layers, and output layer) used for image classification. Studies on SHM have used signal processing techniques combined with CNN to detect and classify damages [32,33].

To use 2D CNN in damage detection and damage classification studies, the sensory data need to be converted into an image for training the network. Ruiz et al. [34] converted the time domain signal data into image texture for classifying different faults of wind turbine blades. Tang et al. [35] produced a composite image by splitting time-series data into multiple segments and adding the visualized time and frequency responses into a 2D array. Time-series sensory data can be converted into 2D time–frequency graphical displays using Short-Time Fourier Transform (STFT) and Continuous Wavelet Transform (CWT). These images can be used for training the CNN algorithms to evaluate the condition of large structures [35] as well as small machines [36].

The effects of print settings and internal hidden geometry on wave propagation characteristics and load sensing were investigated in this study. Seven rectangular bar-shaped specimens were fabricated additively using various print orientations and infills. The wave propagation characteristics were compared with those in a commercially manufactured bar with the same dimensions. Data collection was conducted by using the Surface Response to Excitation (SuRE) method. In order to evaluate the effectiveness of this SHM method in load sensing, the test specimens were loaded at the center with five levels of compressive forces, and CNN was used to estimate the load on the specimen.

## 2. Theoretical Background

An essential aspect of vibration-based SHM studies is the dynamic response to excitation, which has been used in the literature to select damage-sensitive features and conduct statistical analyses as part of the damage assessment process [37]. Guided waves, called lamb waves in thin metal plates, travel in the solid medium for long distances with low attenuation. These waves travel in three modes: symmetric (in plane), anti-symmetric (out of plane), and shear horizontal. It is possible to attach piezoelectric wafer active sensors (PWAS) to the host structure to both excite and measure surface waves. The advantages of piezoelectric elements have made them very popular in SHM studies; they are affordable and lightweight and can be designed in a variety of sizes and geometries. With the SuRE method, an excitation signal is applied to a piezoelectric element to excite guided waves on the surface of the structure, and one or more contact/noncontact sensors are used to monitor the dynamic response to excitation at the desired location. As a result of the viscoelastic behavior of the material, dispersive traveling waves undergo damping, depending mainly on the structure’s properties. With the help of the inverse piezoelectric effect, the waves reaching the sensor are converted to an electric signal which can be displayed in an oscilloscope or recorded by a data acquisition system.

Different signal processing techniques can be used to acquire information regarding the changes in the state of the structure. This can be achieved by identifying critical features that reveal changes in the material state when compared with a reference state. The recorded sensory data in the time domain can be analyzed in the frequency domain by using the Fast Fourier Transform (FFT) algorithm. The FFT calculates the Discrete Fourier Transform (DFT) of a signal in a shorter time. Equation (1) allows the calculation of the DFT for signal *x(n).*

(1) $$X\left(k\right)=\overset{N-1}{\underset{n=0}{\sum }}x\left(n\right)e^{-j\frac{2\pi kn}{N}}$$

where *x(i)* represents the original signal at time *i*, *N* represents the length of the signal, and *X(k)* is a complex number representing the amplitude and phase of a sinusoidal wave. Considering the assumption that the mechanical and electrical properties of piezoelectric elements remain constant during the experiment, any changes in the recorded signal can be referred to as a change in the mechanical properties of the structure. It has been suggested that the Sum of the Square of Differences (SSD) can be used as a damage detection metric in order to calculate the differences in the frequency spectrum between the current state and the reference (also called the baseline) [38].

The FFT spectrum of a signal represents the frequency components of a signal but does not reveal any information about the time components. In order to analyze how the frequency content of a nonstationary signal changes over time, STFT can be used. STFT obtains the spectral characteristics of a signal by calculating the FFT in short time intervals. Spectrograms are time–frequency domain plots created by this transformation [39]. The following equation represents the mathematical expression of the STFT:

(2) $$X_{m}\left(\omega \right)=\sum_{n=-\infty }^{\infty }x\left(n\right)w\left(n-mh\right)e^{-j\omega n}$$

Here, x(n) is the input signal, *w(n)* is the m-point window function, and *h* is the hop size. STFT is based on a fixed-size time-shifted window, with a poor resolution in time or frequency; the window is either small in time, which gives a poor frequency resolution, or wide, which reduces the time resolution.

Continuous Wavelet Transform is a digital processing technique that improves the time and frequency resolution to overcome the limitations of STFT. This technique is based on wavelets which are wave-like oscillations with a zero average, localized in time and that can be asymmetric, non-smooth, and irregular. A signal can thus be divided into a family of wavelets, fundamentally identical but different in their scales and shifts. The CWT uses a size-adjustable window which has a longer window for local areas with low frequencies and a shorter window when the area is localized in the high frequencies. Equation (3) expresses this transformation:

(3) $$T\left(a,b\right)=\frac{1}{\sqrt{a}}\int_{-\infty }^{+\infty }x\left(t\right)\gamma^{*}\frac{\left(t-b\right)}{a}dt$$

where *x(t)* is the input signal, γ is the wavelet function, *a* and *b* are translation and dilation parameters, respectively. The Spectrogram (obtained using STFT) and scalogram (the output of CWT) images acquired from the time-domain sensory data have been implemented as input datasets for the Convolutional Neural Network classification [40].

**Figure 1:**
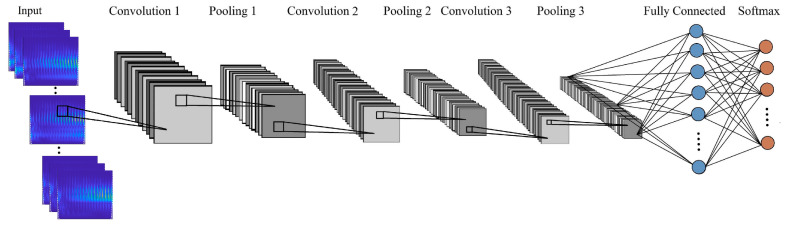
Proposed architecture of the network.

In this study, a two-dimensional CNN was implemented for the classification of the recorded data. The size of the input images was set as 389 × 343 × 3 in width, height, and color channels (red, green, and blue), respectively. An abstract illustration of the CNN architecture is shown in Figure 1; it consisted of three main layers. By applying convolution operations to different filters and the input images, different features were extracted from the input data at the convolutional layer. This layer produced a feature map, which contained detailed information about the input image. This step resulted in a decrease in data width and height, but an increase in data depth. The activation functions were added to the network after the convolution layers in order to introduce non-linearity into the network. Using activation functions helped the network to learn the complex features of the data. There are different types of activation functions in CNNs, but the Rectified Linear Unit (ReLU) is the most common. CNNs consist of a succession of convolution and pooling layers and finish with fully connected and softmax layers. In the pooling layer, the size of the feature map is decreased by summarizing the features, which results in a better computational performance. Among different pooling operations, maxpooling was chosen for this network. In this layer, each patch in the feature map is summarized by selecting the largest value. Fully connected layers output one-dimensional arrays of numbers using the output from the previous layers. Finally, the softmax layer creates a probability distribution for each class, which assigns a probability value to every defined class of data. In this study, the CNN consisted of three building blocks of convolution and pooling layers between the input data and the output. The first, second, and third blocks were composed of 15, 30, and 45 filters, with a kernel size of 3 × 3. The maxpooling layers in each block had filters of size 2  ×  2 with a stride of 2. Table 1 provides the details of the training settings.

## 3. Experimental Setup

**Figure 2:**
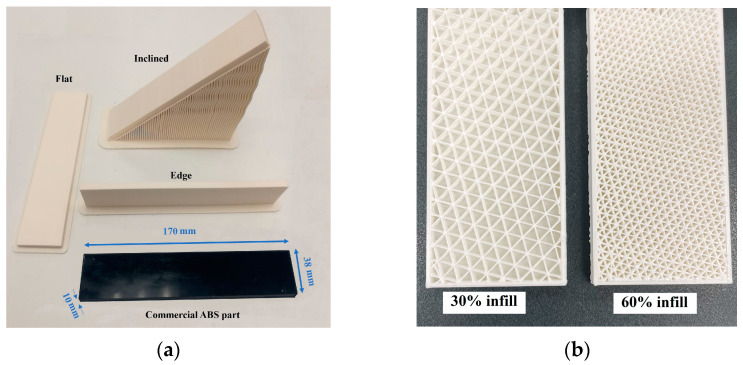
ABS test specimens: (**a**) solid parts; (**b**) parts with infills.

A Funmat Pro 410 3D printer was used to fabricate ABS parts using the Fused Deposition Modeling (FDM) technology at high chamber temperatures to overcome the difficulties of 3D printing ABS parts. To study the effect of the print direction on the wave propagation characteristics and the effectiveness of the SHM method in load sensing on 3D printed polymer parts, eight test bars were prepared with the same dimensions (170 × 38 × 10 mm). The test specimens were categorized into two sets based on their internal geometry characteristics (solid parts and parts with infills). The first set had one conventionally manufactured ABS part which was cut from an ABS plate (manufactured by the extrusion process) and three additively manufactured ABS bars. In Figure 2a, the additively manufactured solid ABS parts with different print orientations are shown, as well as the conventionally manufactured part that was cut from an ABS plate. In the case of Flat and Edge parts, printing was performed in the direction along which the largest and second-largest surfaces of the part were located on the bed. With the help of supports, the Inclined part was fabricated on the largest surface with a 45° angle relative to the print bed. The second test set included four bars with similar dimensions as the solid bars, but with different internal hidden geometries: two parts with 30% rectangular infills, and two with 60% rectangular infills, each with a thickness of 1 mm or 2 mm. Figure 2b shows the internal geometry of the parts with infills. The thickness of the skin is not visible in this photo.

**Figure 3:**
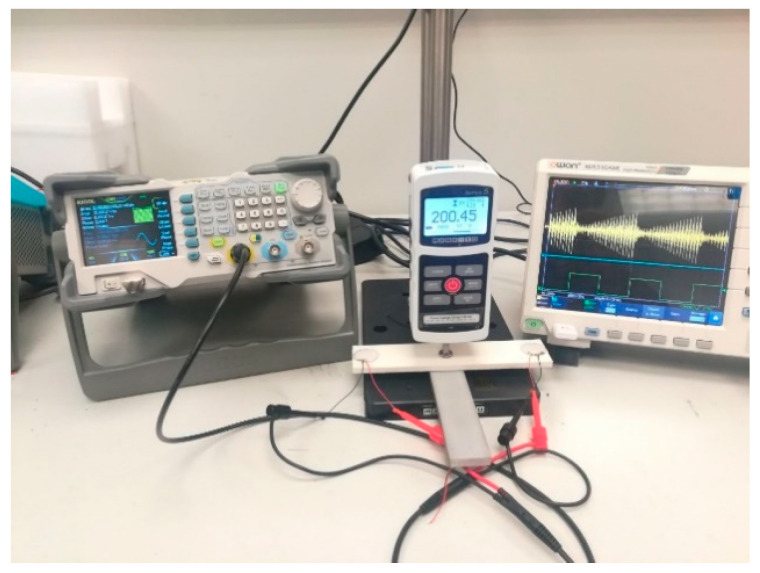
Experimental setup.

Figure 3 shows the experimental setup used in this study for data collection. The SuRE method was implemented to excite the test specimen with surface waves and to monitor the response to excitation. Two piezoelectric disks were permanently attached to each end of the test specimens using the M-Bond 200 adhesive. The parts were excited from one side, and the dynamic response to excitation was monitored on the other side using the other piezoelectric element. Rigol DG1022 was the arbitrary function generator used to generate surface waves on the test specimens, and the Owon XDS3104AE digital oscilloscope was used for recording the sensory data at a sampling rate of 5 MS/s. Three different excitation signals were used for different purposes. A single-pulse excitation signal with 20 μs duration was used for measuring the wave travel time in different parts. To compare the envelopes of the recorded signals in the time domain, a 20 V peak-to-peak sweep sine wave in the 50–150 kHz range was used with a sweep time of 1.0 ms. Multiple-width pulse excitation (MPWE) is the third excitation signal used for load sensing. It consists of 20 consecutive pulses with incremental durations within each step and a 40 μs time delay after each pulse.

In order to evaluate the load estimation accuracy using the SuRE-CNN combination, tests were performed when the parts were in a relaxed state and at four levels of loading. A MARK-10 force gauge was used to hold the center of each part under compression loads of 50 N, 100 N, 150 N, and 200 N. In each loading condition, the experiment was repeated 10 times with one PZT acting as an actuator, and the other as a sensor.

## 4. Results

### 4.1. Surface Waves’ Travel Time

**Figure 4:**
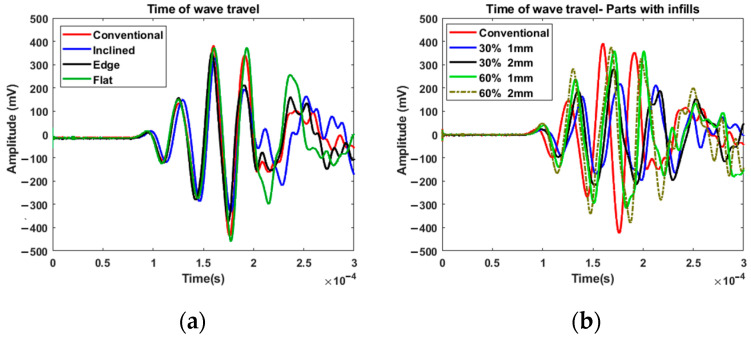
Surface wave travel time in: (**a**) solid parts; (**b**) parts with infills.

In the first experiment, the single-pulse excitation was used for exciting the parts with a short pulse and monitoring the arrival time of the wave to the other end of the test bar. The aim of this experiment was to examine how the print orientation and infill of the 3D-printed specimens affected the wave speed over the ABS test specimens. Figure 4 shows that the first test group, which consisted of solid parts, responded differently to the single-pulse excitation than the second test group. Both plots include the data for the conventionally manufactured part as a reference. As can be observed in Figure 4a, the conventionally manufactured solid parts had a slightly higher wave speed than the additively manufactured ones. It can be observed that the ultrasonic surface waves traveled relatively slowly in the inclined part compared to other solid parts. Figure 4b shows that the surface waves traveled at lower speeds when hidden geometries were designed. It also can be concluded that the surface wave travel speed in 3D printed parts can be increased by increasing surface thickness and infill ratio.

### 4.2. Effect of the Print Settings on Wave Propagation Characteristics

**Figure 5:**
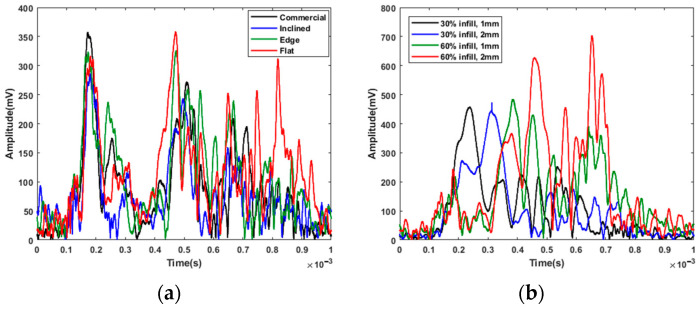
Envelope of the response for: (**a**) solid parts; (**b**) parts with infills.

In the second study, surface waves were excited with a sweep sine wave between 50 and 150 kHz and a sweep time of 0.1 ms. The experiment was performed when the parts were in relaxed condition without any loads on them. Figure 5 presents the envelopes of the recorded signals in the time domain for both test groups. As the frequency of the sweep sine excitation signal increased linearly with time, the observed envelopes are similar to FFT spectrums. It is possible to observe a similar trend for different solid parts, though their responses to excitation differed. It can be seen that the surface waves on the commercially manufactured bar had the highest amplitude compared to those on the 3D printed bars, especially at higher frequencies. Figure 5 shows that at lower frequencies, the monitored signal amplitude for parts with a 30% infill ratio was higher than for parts with a 60% infill ratio, while at higher frequencies, the opposite was true.

### 4.3. External Load Estimation

**Figure 6:**
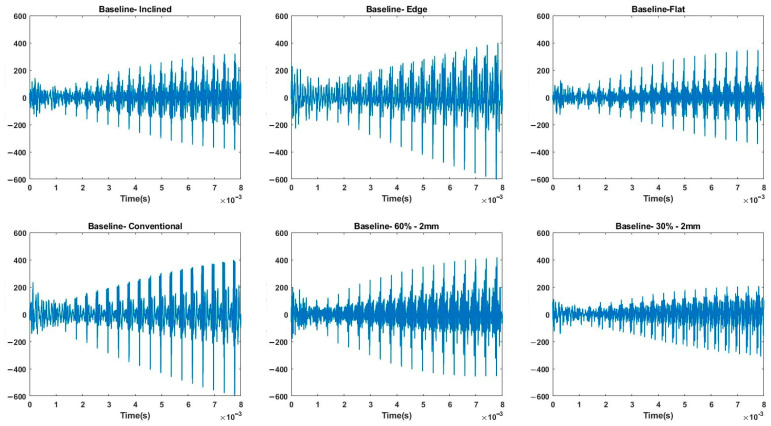
Time domain response to MWPE excitation.

**Figure 7:**
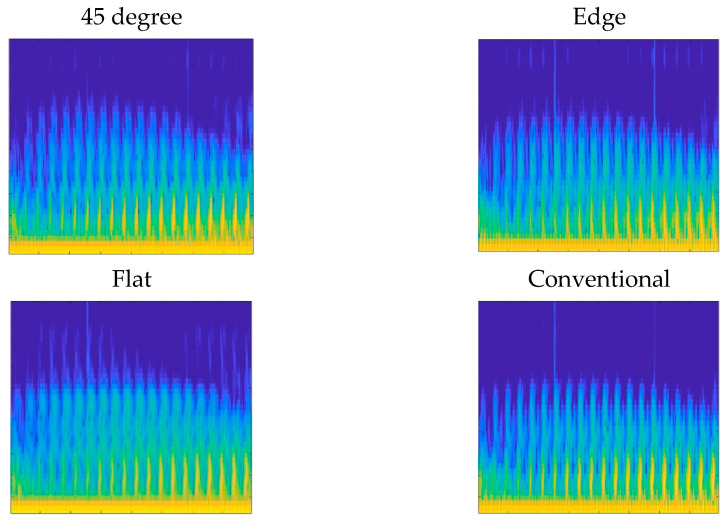
STFT spectrograms of the solid test specimens.

**Figure 8:**
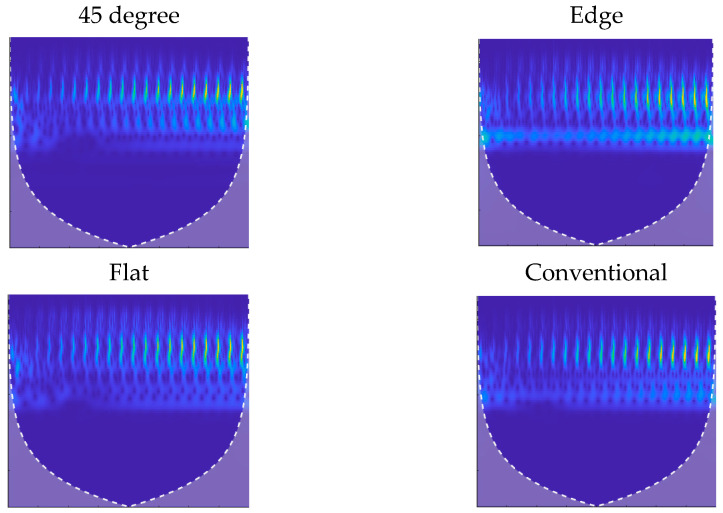
CWT scalograms of the solid test specimens.

For the third study, all parts were subjected to compressive loads from 0 to 200 N in 50 N steps, and the data were analyzed for estimating the applied force. In this experiment, an MWPE excitation signal was applied to the actuator piezoelectric. The MWPE signal consisted of 20 pulses with progressively longer pulses over time. The wave generator was set at the highest voltage level (20 V), and the first pulse lasted for 0.5 μs. There was an increase in pulse widths by 0.5 μs at the following pulses with 400 μs intervals between consecutive pulses. Figure 6 shows the collected sensory data in the time domain when no load was applied on the solid parts and on parts with 30% and 60% infill with 2 mm skin thickness. The fabricated part on the edge showed the most similar time-domain signal to that of the conventionally manufactured part. Spectrograms and scalograms are time–frequency representations derived using STFT and CWT algorithms, respectively. Figure 7 and Figure 8 show the obtained spectrograms and scalograms for solid bars as a sample. To provide the CNN with the most meaningful information, the frequency band was limited between 5 kHz and 2 GHz. The network was trained with 70% of the dataset and tested with 30% of it.

For each study, the CNN was trained through the use of two different image datasets: spectrograms and scalograms. In the first case study, the CNN classified all test bars in a relaxed state without any load applied. For the second case study, the CNN was used to estimate the load applied on the 3D printed solid bars under five different loading conditions. In the third study, the CNN was employed six times to estimate the loading on parts with different infill ratios at five loading conditions using different combinations of data.

**Figure 9:**
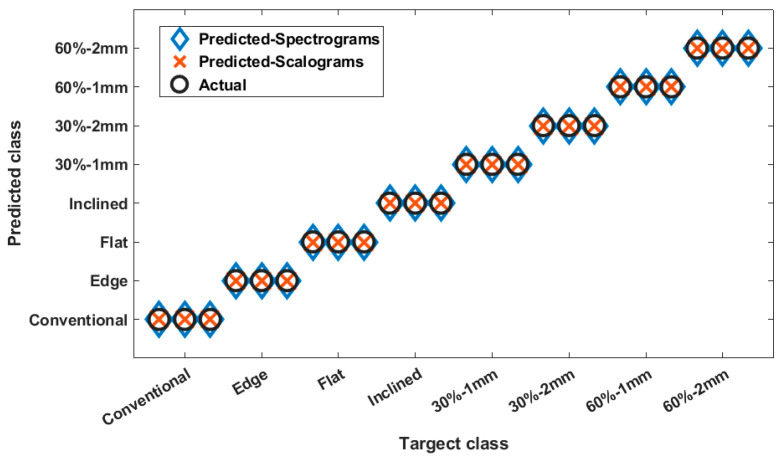
Classification results for all parts under no loading.

The performance of the trained CNNs for the first study is presented in Figure 9. There were eight classes that corresponded to parts with different manufacturing settings, including one conventionally manufactured part, three additively manufactured solid parts, and four additively manufactured parts with infills. The data were collected when no load was applied on the parts. The classification results presented in Figure 9 show that the CNN could distinguish these eight different classes with 100% accuracy, regardless of whether spectrograms or scalograms were used.

**Figure 10:**
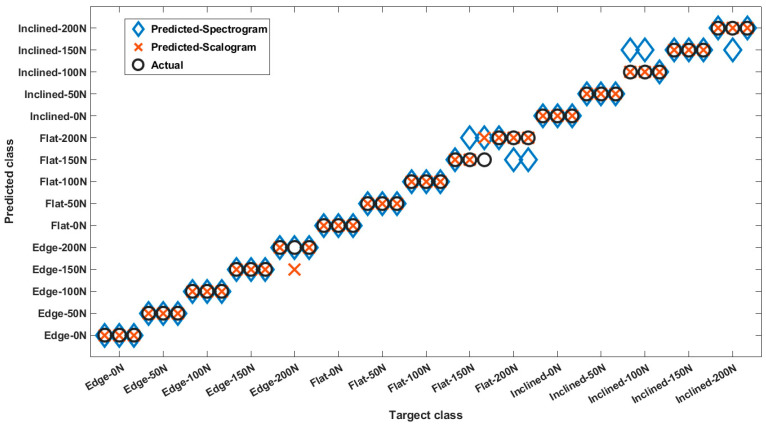
Classification results for solid parts at different loading conditions.

For the second study, two CNNs were trained (using scalogram and spectrogram datasets) to classify 15 classes of the data for the solid parts (Flat, Inclined, and Edge), each at 5 different loading conditions. Based on the results shown in Figure 10, the overall classification accuracy was 84.4% for spectrograms and 95.6% for scalograms. Misclassified cases were within the range of 50 N from the actual test value in both studies.

In the third study, the load for each bar was estimated at five different load levels using data from the 3D printed bars with infills. In total, six CNNs were trained for parts with 30% and 60% infills, and the performance results are presented in Table 2. In comparison to their performance when using spectrograms, the CNNs performed better when trained on scalograms.

## 5. Conclusions

In this paper, surface wave characteristics and wave travel speed were compared between additively manufactured parts and conventionally made ABS bars with the same dimensions of 170 × 38 × 10 mm. Three of the additively manufactured bars were solid and 3D printed at different orientations. Four additional bars were additively manufactured with 30% and 60% infill ratios and 1 mm and 2 mm skin thicknesses. The data were collected using the SURE method, and signal processing techniques were applied to prepare them for the CNN input. For the load sensing task, experiments were performed when a compression load was applied at the center of the specimens at five different levels.

The surface wave speed was almost the same for all the solid parts regardless of the print setting. It took slightly more time for the waves to read the sensor when they moved on the parts with lower infill ratios and/or thinner top surfaces. The envelope of the response to a sweep sine showed a similar behavior in the solid parts, especially at low frequencies. For the parts with a 30% infill ratio, the amplitude of the response was higher than for the parts with a 60% infill ratio at low frequencies, while at higher frequencies the trend was the opposite. 

An MWPE excitation signal was used to collect the data for loading estimation using the CNN. Ten CNNs were trained for the estimation of the loading condition on different parts using spectrograms or scalograms. In the first study, the spectrograms and scalograms of the recorded data were obtained when there was no load on the test specimens. The CNN could classify eight different manufacturing characteristics with 100% accuracy for both scalograms and spectrograms. In the second study, the CNN was used to estimate the applied load on the 3D printed solid parts. We used 70% of the data for 15 different classes (three parts each at five different loading conditions) for training the CNN and 30% for the test. The overall classification accuracy was 84.4% when using spectrograms and increased to 95.6% when using scalograms. Misclassified cases in both studies were within the range of 50 N from the actual values. In the third task, six different CNNs were trained to quantify the loading when the test bars had infills. The load estimation accuracy was within the range of 83–100%. This study also showed that the performances of the CNNs trained with the scalograms were slightly higher. 

These results indicate that very small surface wave speed and attenuation characteristics adjustments may be made by controlling the infill-related parameters during the additive manufacturing process. The combination of the SuRE method and the CNN can be used to identify the manufacturing details or quality of a part. Even when combining data from parts made with different build orientations, the CNN estimated the load levels with good accuracy. In all the studies, the CNN estimation accuracy ranged from 83% to 100%, which is satisfactory for many applications. In this study, the classification based on scalograms showed better results, with a small margin, compared with the results obtained when using spectrograms. The calculation of scalograms is much faster and more efficient than that of spectrograms and may be preferred in industrial applications.

## Figures and Tables

**Table 1:** Training settings of the CNN.

Solver	Initial Learn Rate	Max Iteration	Shuffle
sdgm	0.0001	150	Every-epoch

**Table 2:** Classification results for 3D printed parts with infills.

Parts	Inputs	No. of Classes	Training Cases	Testing Cases	Errors	Accuracy
30% infills	Spectrogram	10	70	30	5	83.3%
	Scalogram	10	70	30	2	93.3%
60% infills	Spectrogram	10	70	30	3	90.0%
	Scalogram	10	70	30	0	100%
30% and 60% infills	Spectrogram	20	140	60	10	83.3%
	Scalogram	20	140	60	5	91.7%
